# Role of specific immunoglobulin-E in chronic rhinosinusitis: Its clinical relevance according to nasal challenge test^☆^

**DOI:** 10.1016/j.waojou.2024.100953

**Published:** 2024-10-07

**Authors:** Jorge Sánchez, Leidy Álvarez, Juan Bedoya, Daniel Peñaranda, Gustavo Vanegas, Carlos Celis, Edison Morales, Elizabeth García, Augusto Peñaranda

**Affiliations:** a Group of Clinical and Experimental Allergy, Hospital “Alma Mater de Antioquia”, University of Antioquia, Medellín, Colombia; b Group "ciencias de la vida y de la salud escuela de graduados", CES university, Medellín, Colombia; c Otorhinolaryngology Service, University of Antioquia, Medellín, Colombia; d “Fundación Universitaria Ciencias de la Salud”, Otorhinolaryngology Service, Bogotá, Colombia; e Faculty of Medicine, Pontificia Universidad Javeriana, Bogotá, Colombia; f Allergology Unit, Medellín, Colombia; g Otorhinolaryngology Medical Surgical Unit (UNIMEQ-ORL), Bogotá, Colombia

**Keywords:** Allergy, Atopy, Dupilumab, Immunoglobulin E, Immunotherapy, Nasal challenge test, Nasal provocation test, Omalizumab, Rhinosinusitis

## Abstract

**Background:**

Guidelines for chronic rhinosinusitis (CRS) propose total IgE and eosinophils as important biomarkers to identify type-2 inflammation. Despite the fact that specific IgE (sIgE) have been identified as a clinical predictor in some type-2 diseases for different clinical outcomes, its role in CRS has yet to be explored in detail.

**Objetive:**

To describe systemic and local sIgE in CRS and explore its possible association with clinical outcomes using nasal challenge tests (NCT).

**Methods:**

In CRS patients, we measure total IgE, serum sIgE (SsIgE) and nasosinusal sIgE (NsIgE) against 9 allergenic sources; Der p, Der f, Blo t, Can f, Fel d, Per a, grasses, Staphylococcus enterotoxin A, and B. NCT was done using the allergen with the higher sIgE prevalence (Der p).

**Results:**

A total of 174 patients were included. Prevalence of SsIgE was 52.8% and NsIgE 46.5%; Der p was the principal allergen for SsIgE and NsIgE. The presence of nasal polyps, asthma comorbidity, NSAID hypersensitivity, and hyposmia, were significantly associated with the presence of SsIgE and NsIgE but not with total IgE. NCT-Der p was performed in 73 CRS patients, being positive in 33 (45.2%). SsIgE have the best diagnostic accuracy (79.4%) to predict NCT results (NsIgE 67.5% total IgE 52%).

**Conclusion:**

Specific IgE is a better biomarker in CRS than total IgE. Patients with clinically relevant SsIgE have a pheno-endotype associated with different clinical outcomes. Considering the clinical relevance of SsIgE demonstrated by NCT, interventions like allergen immunotherapy in CRS must be study.

## Graphical abstract

**Figure:**
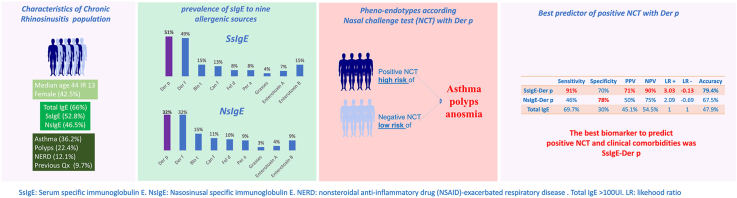

## Introduction

Chronic rhinosinusitis (CRS) is a high impact disease that effects approximately 3–15% of population.^1^, ^2^, ^3^ It consists of inflammation in the mucosa of the nose and paranasal sinuses that leads to a set of symptoms with a high individual impact on quality of life, work activities, and social activities, but also at a collective level in the consumption of health resources and decreased productivity.^4^, ^5^, ^6^, ^7^

In CRS there are different endotypes defined by the underlying inflammation profile, with type 2 inflammation (T2-inflammation) being the most studied because it is associated with more severe symptoms such as the coexistence of nasosinusal polyps, severe asthma, and nonsteroidal anti-inflammatory drug (NSAID)-exacerbated respiratory disease (NERD).^8^, ^9^, ^10^, ^11^, ^12^ Studies carried out in populations in Europe, North America, and Asia show that patients with CRS from many parts of the world share certain common clinical characteristics, but also present differences, such as the frequency of polyposis, the age at onset, and the prevalence of inflammatory endotypes.^13^, ^14^, ^15^ At the moment, there are few studies evaluating the characteristics of CRS in tropical regions such as countries in Latin America and the African continent,^16^^,^^17^ being in most cases focused on the clinical characteristics (phenotypes) but not on the endotypes.

Among the clinical biomarkers that allow us to identify the presence of T2-inflammation, are specific immunoglobulin E (sIgE) in blood and in the sinonasal mucosa. Several studies in allergic rhinitis (AR) and asthma have shown that the measurement of sIgE is useful for defining personalized management such as allergen immunotherapy and to predict some clinical outcomes.^18^^,^^19^ CRS guidelines like EPOS and EUFOREA recommend using total IgE and serum eosinophils as biomarkers of T2-inflammation in CRS,^1^^,^^20^ but according to these guidelines the role of sIgE in CRS is still controversial.

In this study, we describe the characterization of the phenotypes and endotypes of patients with CRS according sIgE in a population located in the tropical region and we evaluated for the first time the diagnostic performance and clinical relevance of sIgE in blood and in the sinonasal mucosa according to the results of the nasal challenge with allergens. Additionally, we explored the clinical application of serum sIgE (SsIgE) and nasosinosal sIgE (NsIgE) as predictors biomarkers for common clinical comorbidities in CRS (nasosinusal polyps, severe asthma, and NERD)

## Methods

### Study design

Descriptive cohort study with exploration of possible relations between molecular and clinical components. In this article we present the clinical characterization and the specific IgE (sIgE) response to environmental allergens of the RRReNet cohort (Rhinitis and Rhinosinusitis Research Network). Patients older than 18 years with primary CRS were included according to the clinical criteria proposed in the EPOS guidelines^1^ and confirmed by tomography or sinonasal endoscopy. Patients with a medical condition that could affect the interpretation of the clinical scales (eg, congenital or post-trauma nasal cavity anomaly, peripheral neuropathies, primary ciliary atrophy, cystic fibrosis, selective immunodeficiencies) or with secondary CRS-associated diseases such as ANCA-related vasculitis, were excluded. Patients who were receiving drugs that could affect the initial immunological evaluation were excluded, but if they received the biologics after the recollection of the baseline biological samples they were not excluded. Sociodemographic and clinical information of the patients was collected through clinical assessment tools; SNOT22 (Sino-Nasal Outcome Test),^21^ TNSS (Total Nasal Symptom Score),^22^^,^^23^ VAS (Visual analogue scale)^1^ for olfaction assessment, and ACT (Asthma Control test). Immunological markers were analyzed using blood and sinonasal mucus samples. NCT were done with the most prevalent allergenic source. Anosmia was defined as a VAS ≤2 points.

### Definition of groups

Atopy was defined as the presence of SsIgE for at least 1 allergen. Patients with CRS were divided into groups according to the present or not of SsIgE. A rhinitis group and healthy group were recruited to exploratory comparisons of the frequency of atopy in CRS and its clinical relevance according NCT. The 2 groups had similar sociodemographic characteristics to the CRS group (eTable 1).

### Measurement of IgE antibodies

All patients with CRS underwent a skin prick test (SPT) with a panel of 24 aeroallergens (eTable 2). Serological and mucus sIgE measurement were performed for the allergenic sources more frequent in SPT; *Dermatophagoides pteronyssinus* (Der p), *Dermatophagoides farinae* (Der f), *Blomia tropicalis* (Blo t), *Canis familiaris* (Can f), *Felix domesticus* (Fel d), *Aspergillus fumigatus* (Asp f), Grasses spp. The SsIgE and NsIgE against Staphylococcus aureus enterotoxins (A and B) were measured in all patients.

SsIgE and NsIgE were measured by ImmunoCAP system according to previously described procedures using 0.35 kU_A_/ml as cut-off point.^25^ Sera yielding sIgE levels above 100 KU_A_/ml were preliminarily diluted (1:5) to maintain the test within the dynamic range. For the collection of nasosinusal mucus sample we perform a lavage following the method recommended by Naclerio with some adaptations.^21^^,^^25^^,^^27^ For the measurement of NsIgE a calibration curve with samples from 20 healthy subjects was made using the ImmunoCAP system; the value of 0.12 kU_A_/ml was considered as the cut-off point according to the mean and two standard deviations.

### Nasal challenge test

The evaluation of clinical relevance based on NCT was performed according to international recommendations.^25^^,^^29^ The choice to perform the NCT with *Dermatophagoides pteronyssinus* (Der p) was based on the fact that it was the most frequent allergenic source in the study and this is in line with previous studies in the reference population^24^, ^25^, ^26^, ^27^, ^28^ but also in in others CRS populations.^30^^,^^31^ The Der p extract (Laboratory Inmunotek, Spain) at a concentration of 10,000 UB/mL was applied with a nasal spray in a measured dose of 100 ml/puff in each nostril. Previously, the presence of non-specific nasal hyperreactivity was ruled out by performing the same procedure with saline solution. The result of the test was evaluated objectively by performing acoustic rhinometry with a reduction greater than 20% considered positive, and with the Lebel score.^25^^,^^29^^,^^32^ To define a positive NCT, the criteria proposed by the “European Academy of Allergy and Clinical Immunology” (EAACI) was used.^29^ Patients who received biologic therapy (Benralizumab, Dupilumab, Mepolizumab, Omalizumab) after the first NCT, received an invitation to a second measure of sIgE and NCT once they received the biologic therapy for six months.

### Bias control

#### Selection bias

Diagnosis of CRS was done by expert physicians in the identification of the disease (allergists, otolaryngologists, pulmonologists) using clinical history and confirmed by tomography or nasolaryngoscopy in accordance with the EPOS guidelines. Due to the type of design, we recruit patients with any degree of severity, from primary care centers or specialized centers in allergology and/or otorhinolaryngology which reduces the risk of prevalent cases and other selection biases.^33^

#### Information bias (measurement bias)

The variables to be measured are clinical and laboratory tests. Staff physicians were trained before the start of the study to unify concepts regarding the measurement and the way to carry out the questionnaires. In the case of laboratory variables, these were evaluated in duplicate, and the laboratory personnel were unaware of the characteristics of the patients. Clinical relevance of IgE sensitization was evaluated with acoustic rhinometry reducing operator bias.

### Statistical analysis

Because we did not have an a priori hypothesis, we did not pre-establish a sample size. We carried out a convenience sampling for a period of one year. For the descriptive analysis, absolute frequencies, relative frequencies, and summary measures, such as the median or the interquartile range, were used. The criteria of normality of clinical and laboratory variables were evaluated by Shapiro-Wilk test indicating a non-normal distribution for most variables. To establish the relationship between the results of the NCT and sIgE with respect to the study groups, the Pearson chi-square test of independence were applied or Fisher's exact test when the amount of data in the cells was less than 5. Mann-Whitney *U* test was used for comparisons between continuous variables. A *p* value ≤ 0.05 was considered statistically significant. For the correlation between the levels of NsIgE and SsIgE, the spearman correlation coefficient was used. Indicators of diagnostic accuracy for Der p SsIgE and NsIgE were measured using NCT as gold standard test. The statistical program SPSS version 26 and Prism version 9 were used for statistical analysis.

### Bioethical considerations

The study protocol was approved by the institutional ethics committee (code IN48-2021 act #179, Hospital “Alma Mater de Antioquia”) and is in line with the Helsinki declaration. Each of the participants signed a informed consent.

## Results

### General characteristics

A total of 174 patients with CRS were recruited with a predominance of male sex (57.5%) over female (42.5%). Median age was 44 years. Asthma was the most frequent comorbidity (36.2%) followed by sinonasal polyps (22.4%), (NERD) (12.1%), and anosmia (10.3%) (Table 1). The median of SNOT22 was 24 points. Among patients with asthma ACT median was 18 points.

**Table 1:** General characteristics.

Characteristics	Categories	Study groups			*p*
		Global n = 174	No atopy n = 82 (48.2%)	Atopy n = 92 (52.8%)	
**CRS duration**	Months	Me 12 (RI 28)	Me 12 (RI 27)	Me 12 (RI 28)	0.780
**Sex**	Female	74 (42.5%)	36 (43.9%)	38 (58.7%)	0.760
	Male	100 (57.5%)	46 (56.1%)	54 (41.3%)	
**Age group**		Me: 44 (RI: 13)	Me: 43 (RI: 14)	Me: 44 (RI:13)	0.810
**Comorbidities**	Asthma	63 (36.2%)	19 (23.2%)	44 (47.8%)	**0.001**
	NERD	21 (12.1%)	5 (6.1%)	16 (17.4%)	**0.034**
	Current polyps	39 (22.4%)	15 (18.3%)	24 (26.1%)	0.275
	Anosmia	18 (10.3%)	8 (9.8%)	10 (10.9%)	1
	Surgery for polyps	17 (9.7%)	3 (3.7%)	14 (15.2%)	**0.011**
**Symptoms**	SNOT22	Me: 24 (RI: 19)	Me: 21 (RI: 16)	Me: 32 (RI:23)	**<0.001**
	TNSS	Me: 7 (RI: 4)	Me: 6 (RI: 5)	Me: 7 (RI: 3)	**0.027**
	VAS	Me: 4 (RI: 4)	Me: 3 (RI: 4)	Me: 4 (RI: 4)	**0.027**
	ACT	Me: 18 (RI: 6)	Me: 20 (RI: 7)	Me: 18 (RI: 6)	0.625
**Atopy**	IgE monosensitization	37 (21.2%)	No apply	37 (40.2%)	No apply
	IgE polysensitization	55 (31.6%)	No apply	55 (59.8%)	No apply
**Biomarkers**	Total IgE	Me: 123 (RI 121)	Me: 106 (RI:134)	Me: 127 (RI: 111)	0.108
	sIgE Der p	89 (51.1%)	0	89 (96.7%)	No apply
	sIgE Der f	86 (49.4%)	0	86 (93.5%)	No apply
	sIgE Blo t	27 (15.5%)	0	27 (29.3%)	No apply
	sIgE Dog	24 (13.8%)	0	24 (26.1%)	No apply
	sIgE Cat	15 (8.6%)	0	15 (16.3%)	No apply
	sIgE cockroach	15 (8.6%)	0	15 (16.3%)	No apply
	sIgE grasses	8 (4.6%)	0	8 (8.7%)	No apply
	sIgE enterotoxin A	13 (7.5%)	0	13 (14.1%)	No apply
	sIgE enterotoxin B	27 (15.5%)	0	27 (15.5%)	No apply
	snIgE Der p	28 (32.6%)	4 (7.8%)	24 (68.6%)	**<0.001**
	snIgE Der f (total 51:36)	27 (31.4%)	2 (3.9%)	25 (71.4%)	**<0.001**
	snIgE Blo t	13 (15.1%)	0	13 (37.1%)	**<0.001**
	snIgE Dog	10 (11.6%)	1 (2%)	9 (25.7%)	**0.001**
	snIgE Cat	9 (10.5%)	1 (2%)	8 (22.9%)	**0.003**
	snIgE cockroach	8 (9.3%)	2 (3.9%)	6 (17.1%)	**0.058**
	snIgE grasses	3 (4.7%)	0	3 (8.6%)	**0.064**
	snIgE enterotoxin A	4 (4.7%)	1 (2%)	3 (8.6%)	0.3
	snIgE enterotoxin B	8 (9.3%)	1 (2%)	7 (20%)	**0.007**

Sociodemographic characteristics of the patients. Atopy was defined as the presence of IgE in serum for at least one allergen. sIgE: specific IgE in serum. SnIgE: specific IgE in nasosinusal mucus. In bold p values under 0.05.

### Presence of systemic and local specific IgE

**Fig. 1:**
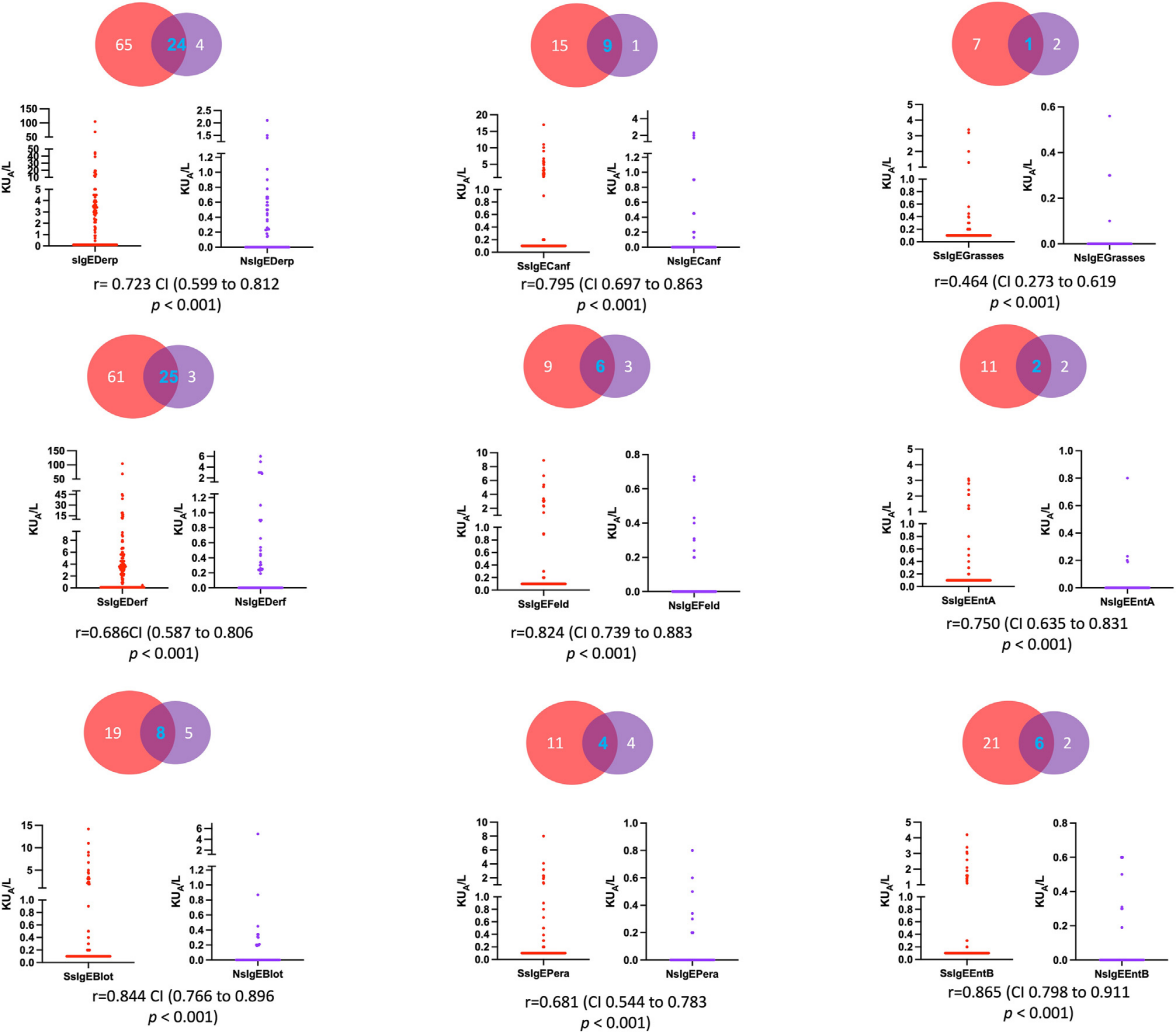
**sIgE in blood and nasosinusal mucus.** Levels of and nasosinusal mucus (NsIgE). In red circles, the patients with serum specific IgE (SsIgE) for each of the allergens. In the purple circle, the nasosinusal mucus specific IgE for each of the allergens

Out of 174 patients with CRS, 92 (52.8%) had SsIgE and 115 (66%) total IgE over 100 kU/ml (Table 1). From 86 nasosinusal mucus samples, 40 (46.5%) had NsIgE. A total of 88 patients did not accept the procedure for mucus samples: we compared the sociodemographic and clinical characteristics between patients who agreed to donate the nasal mucus sample and those who did not according to frequencies and a multilevel analysis: we did not find significant differences between the groups. The order of allergenic sources sensitization frequency was the same for NsIgE and SsIgE; the most prevalent were Dermatophagoides spp., followed by *Blomia tropicalis* and dog. The frequency of NsIgE was lower than SsIgE and most patients with NsIgE had also SsIgE (Table 1 and Fig. 1). There was a correlation between NsIgE and SsIgE for the 9 allergenic sources evaluated (Fig. 1) ranging from moderate to high. The presence of asthma, NERD, and severe symptoms (SNOT22, TNSS and VAS) were significantly more frequent in CRS patients with atopy (Table 1). Although current nasal polyposis was not associated with atopy, previous polyps’ surgeries were higher in CRS with SsIgE.

### Clinical relevance of SsIgE and NsIgE

**Fig. 2:**
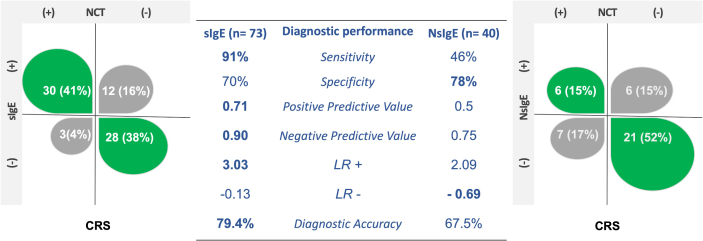
**Diagnostic performance in CRS patients.** Diagnostic performance of patients according to positive or negative SsIgE or NsIgE based in NCT results.

A total of 73 NCT-Der p were done; 33 (45.2%) were positive (Table 2). Asthma, polyps, and anosmia were associated with NCT-positive result. Severity of CRS according to SNOT22 was higher in positive-NCT group but not statistically significant (p 0.06). A higher number of patients with positive-NCT had NsIgE. During the follow-up, seven patients received biologic therapy (Dupilumab n = 5 and omalizumab n = 2). To evaluated change in IgE response, after 6 months with biological therapy we did a second measured of SsIgE and NsIgE for Der p (Fig. 2). We observed a significant reduction in sIgE levels in all patients. Four patients with a previous NCT-positive accept a second NCT after 6 months with biologic therapy; 2 of them tolerated the new NCT with Der p. The odds ratio of each diagnostic tool for different clinical phenotypes was present (Fig. 5).

**Table 2:** Characteristics of patients according to nasal challenge test results.

Characteristics	Categories	NCT		*p*
		Negative n = 40 (54.7%)	Positive n = 33 (45.2%)	
**Sex**	Female	19 (47.5%)	14 (42.5%)	0.665
	Male	21 (52.5%)	19 (47.5%)	
**Age group**		Me: 44 (RI: 13)	Me: 44 (RI:13)	0.910
**Comorbidities**	Asthma	10 (25%)	17 (51.5%)	**0.02**
	NERD	4 (10%)	7 (21.2%)	0.207
	Polyps	7 (17.5%)	16 (48.4%)	**0.005**
	Anosmia	3 (7.5%)	9 (27.2%)	**0.023**
	Surgery for polyps	4 (10%)	5 (15.1%)	0.723
**Symptom**	SNOT22	Me 23 (RI 19)	Me 34 (RI 36)	**0.06**
	TNSS	Me 6.5 (RI 5)	Me 8 (RI 4)	0.168
	VAS	Me 4 (RI 4)	Me 6 (RI 5)	0.179
	ACT	Me 18.5 (RI 5)	Me 18 (RI 6)	0.802
**Atopy**	SsIgE	13 (32.5%)	30 (90.9%)	**<0.001**
	NsIgE (n 40)	8/27 (29.6%)	10/13 (76.9%)	**0.007**
	IgE polysensitization	1 (33.3%)	2 (66.7%)	No apply
**Biomarkers**	Total IgE	Me 123.5 (RI 141)	Me 125 (RI 153)	0.914
	sIgE Der p	Me 0.1 (RI 5)	Me 4.3 (RI 4)	**<0.001**

Sociodemographic characteristics of the patients. Atopy was defined as the presence of sIgE in serum for at least one allergen. sIgE: specific IgE in serum. SnIgE: Specific IgE in sinonasal mucus. In bold p values under 0.05.

### Diagnostic performance of SsIgE and NsIgE

**Fig. 3:**
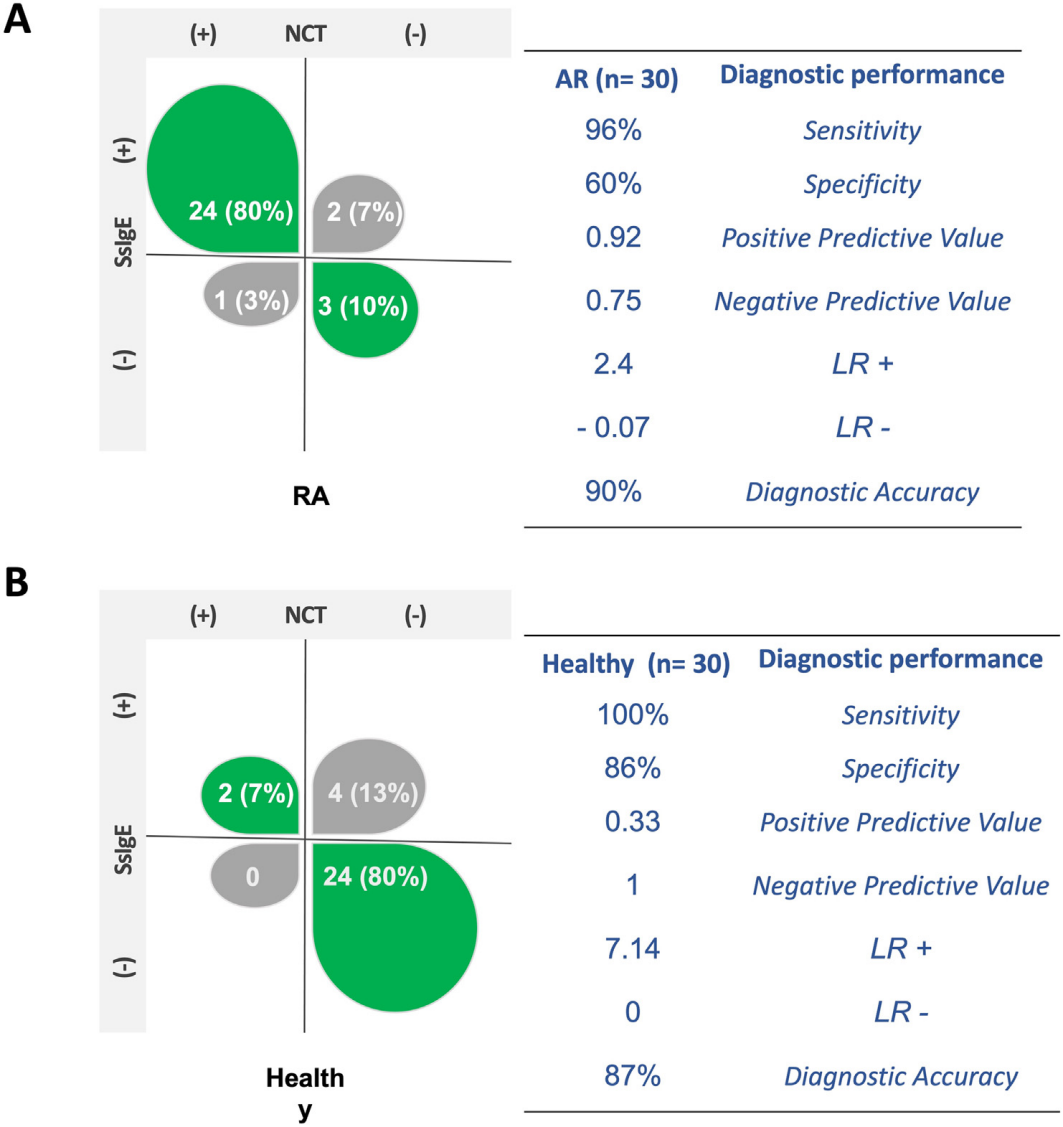
**Diagnostic performance in Allergic rhinitis and healthy subjects.** Diagnostic performance according to positive or negative SsIgE or NsIgE based in NCT results. (A) allergy rhinitis (AR), (B) healthy subjects.

As an exploratory analysis of the utility of atopy as biomarker in CRS we evaluated the diagnostic performance of SsIgE and NsIgE using NCT as gold standard (Fig. 3). The diagnostic sensitivity of SsIgE was 91% and for NsIgE was 46%. The correct identification of negative-NCT patients was similar between SsIgE and NsIgE with a diagnostic specificity of 70% and 78% respectively. Predictive values were higher for SsIgE than NsIgE in CRS patients. The diagnostic performance of total IgE (Sensitivity 69.7%, specificity 30%, PPV 45.1%, NPV 54.5%, LR(+) 1, LR(−) 1, diagnostic accuracy 47.9%), was lower than sIgE.

**Fig. 4:**
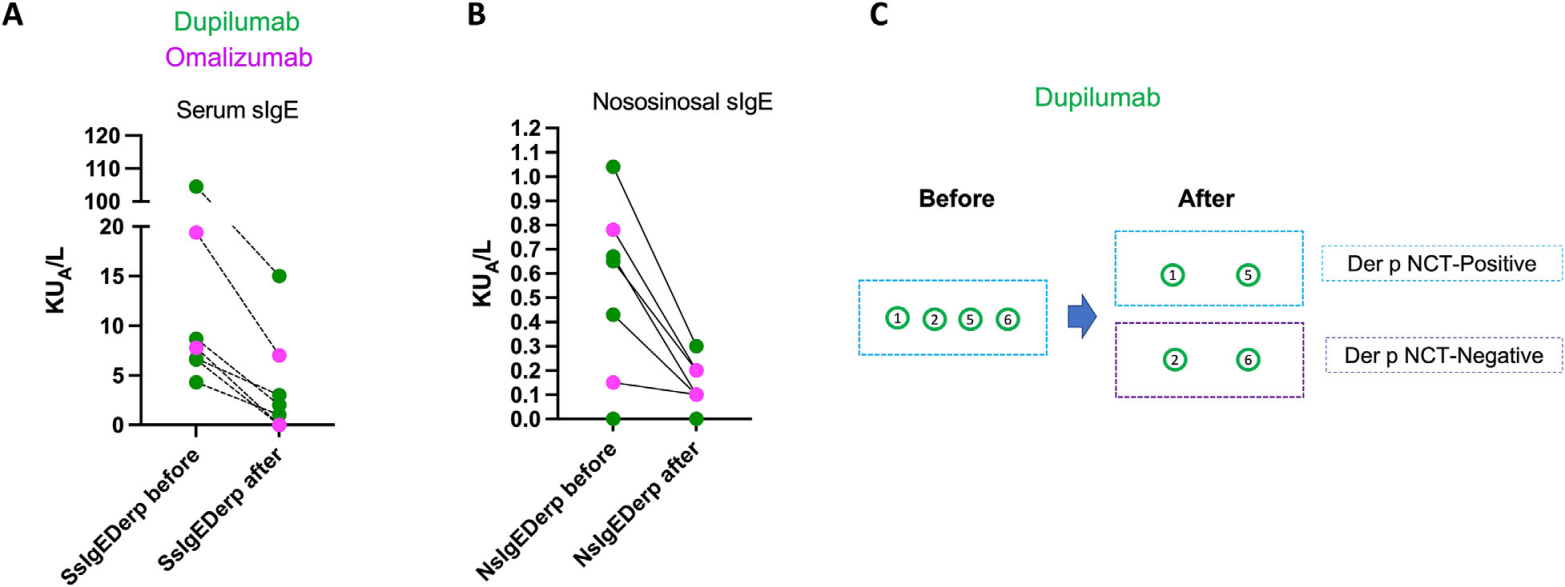
**NCT and sIgE before and after biologic therapy.** Seven patients received biologic therapy (Dupilumab n = 5 and omalizumab n = 2). In green circles patients with dupilumab and in fuchsia patients with omalizumab (n = 2). Change in levels of SsIgE (A) and NsIgE after six months with biological therapy (B). Patients with a second challenge test (C).

When compared the frequency of positive-NCT between CRS and AR, were higher in AR group (80%) than in CRS (41%) (Fig. 3, Fig. 4). The usefulness of the SsIgE to identify positive-NCT patients (sensitivity) was similar between RA group (96%) and CRS (91%).

## Discussion

Frequency of sIgE sensitization (atopy) among CRS patients diverges widely among populations.^1^, ^2^, ^3^ In CRS the prevalence of atopy and the role of IgE in CRS pathogenesis has been studied^34^, ^35^, ^36^ but little information is available about the clinical relevance of sIgE using NCT in CRS and the role of sIgE as predictor biomarker of clinical outcomes. According to EPOS and EUFOREA guidelines the role of atopy in CRS continues to be controversial^1^^,^^20^ and according to these guidelines the level of evidence is poor based on the available data. The same guidelines recommends the use of total IgE and serum eosinophils for identification of T2 inflammation. Although total IgE may be useful to identify T2 inflammation, according to our results its predictive capacity for clinical outcomes in CRS is lower compared to sIgE.

As part of T2 inflammation, atopy could be a prognostic biomarker in CRS of disease activity, polyp recurrence, and risk of NERD. Nevertheless, it is necessary to confirm this results in other populations, especially where predominate IgE sensitization with other allergens different to house dust mites.

**Fig. 5:**
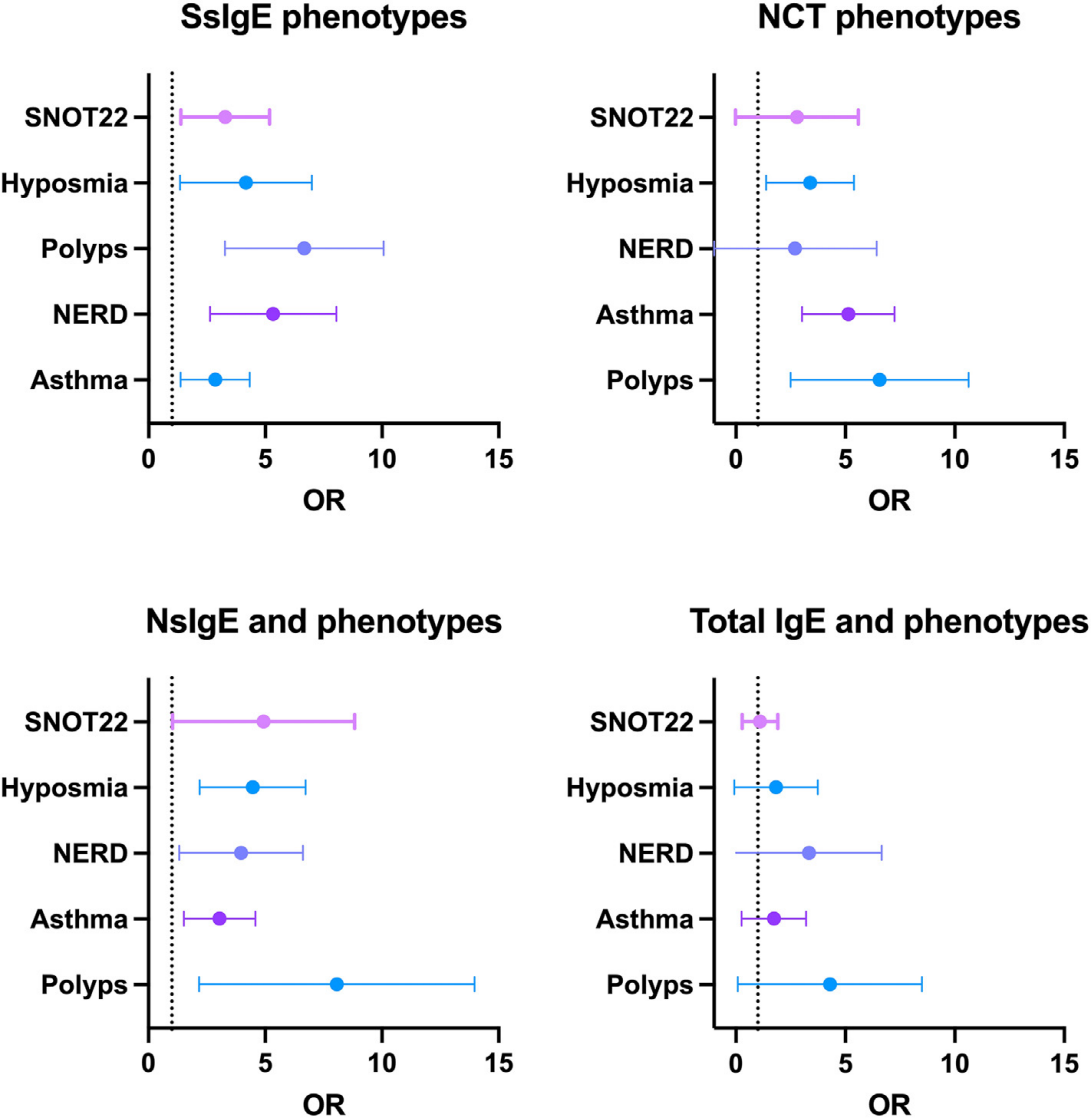
**Odd ratio (OR) for SsIgE (Serum specific IgE), NsIgE (Nasal specific IgE), Total IgE, and NCT (Nasal Challenge test).** Polyps was defined as polyps in any moment (Actual nasal polyps and previous polyps' surgeries)

Similar to previous studies for allergic rhinitis and allergic asthma,^37^^,^^38^ we observed that Dermatophagoides spp., was the main source of IgE sensitization in CRS; we observed that 52% of CRS patients had atopy and this group had a higher frequency of asthma, NERD, and the presence of polyps, which supports its usefulness as a potential biomarker to identify different endophenotypes. Although current nasal polyposis was not associated with atopy, previous polyps’ surgeries were higher in CRS with SsIgE. When evaluating the risk of nasal polyposis at any time (Fig. 5), we observed that this clinical variable was associated with SsIgE, NsIgE and NCT.

Challenge tests are currently the gold standard for allergy diagnostic.^29^^,^^39^ It has been frequently used in CRS to confirm the diagnosis of NERD.^40^^,^^41^ On the other hand few studies used NCT in CRS; Baroody et al,^42^ used NCT to evaluated inflammation in CRS demonstrating that NCT-allergen produce an increase in the eosinophilic infiltrate and in histamine levels in CRS patients, but in this study the relationship with different clinical outcomes was not evaluated. Additionally, in murine models it has been shown that sinonasal symptoms that occur after exposure to an allergen are mainly mediated by IgE.^43^ These observations and our results support that sIgE in CRS is more than an epiphenomenon. Additionally, we observed that sIgE and NCT could be useful for the identification of pheno-endotypes with high or low risk for different clinical outcomes.

The relevance of sIgE in the pathogenesis of CRS is also supported for our exploratory results with biologic therapies; the reduction of SsIgE and NsIgE after six months with dupilumab or omalizumab and the NCT-allergen tolerance in some patients after dupilumab, supported that sIgE is part of the pathogenesis of CRS symptoms but also it could be a potential biomarker for the selection of biological therapy in these patients. These hypothesis must be confirmed with a higher number of patients.

To our knowledge, only 1 study has previously evaluated the correlation between systemic and local allergen IgE sensitivity in CRS. At the sinonasal tissue level, Edwards T et al,^44^ observed a correlation between systemic and local IgE levels for 15 allergens ranging from weak to strong in 15 patients with CRS subtype central compartment atopic disease (CCAD). Similarly, we observed a moderate to strong correlation between the levels of SsIgE and NsIgE for 9 allergenic sources. The lower frequency of IgE in nasosinusal mucosa compared to serum levels may be due to technical difficulties in allergen detection, but the results observed in the RA group and in the Healthy group indicate that this is unlikely. Therefore, our hypothesis is that the presence of NsIgE is fluctuating, perhaps secondary to the intensity of the allergen exposure at the time when the sample is taken.

Calús et al,^45^ performed challenge tests in grass pollen-sensitized CRSwNP patients and in a group of patients with allergic rhinitis. In the group of CRS with polyps, 6/12 patients had a positive challenge test during the NCT, while in the group of grass pollen allergic rhinitis patients, 12/12 patients had a positive challenge during the test. Like Calús, we observed that atopy was a good predictor of a positive challenge test in CRS, although it was lower than that observed in patients with rhinitis. We believe that this is because CRS is a disease that shares mechanistic processes with allergic rhinitis but also has independent mechanisms.

For a practical point of view and in search of precision medicine, according with our results a management proposal would be that patients with CRS and a positive atopy test undergo a NCT to confirm whether this mechanism is clinically relevant. The correct identification of CRS patients with IgE clinically relevant presents potential prognostic and therapeutic advantages. We observed that the CRS patients with positive-NCT were the patients with the highest risk of asthma, polyposis, and NERD, therefore, carrying out the challenge test allows us to identify the most severe patients that may require specific therapies. In other allergic diseases (asthma, conjunctivitis, rhinitis, and atopic dermatitis) when IgE sensitization is clinically relevant, allergen immunotherapy (AIT) has been shown to be effective. In CRS there is little information on the clinical impact of AIT; some studies suggest positive results,^46^, ^47^, ^48^, ^49^ but none of them have a comparison group or a randomized design. Nevertheless, our results, support the importance of future studies in this point. The impact of environmental control measures in CRS has been little studied. Some irritants such as cigarette smoke and air pollution have been associated with exacerbation of symptoms and decreased response to drug treatment;^50^, ^51^, ^52^ however, we were unable to find studies that evaluated the impact of environmental allergen control on CRS. Studies aimed at evaluating the magnitude of the effect of these two therapies are required to establish their usefulness in clinical practice.

The study has several strengths and weaknesses. The control of measurement and selection biases allows for reliable results. The number of participating patients and the collection of patients not only in specialized centers but also in primary care centers makes it possible to assess the frequency of atopy not only in the most severe CRS patients. To our knowledge, this is the first study that calculate the diagnostic performance of Der p-sIgE in CRS. However, among the weaknesses of the study is the lack of evaluation through nasal challenges of other allergenic sources. It would also have been interesting to explore the impact on NCT of other biological therapies and to explore the interaction of IgE with other mechanisms, for example, the levels of eosinophils in the nasosinusal mucosa. Although the presence of polyps was confirmed by tomography or sinonasal endoscopy, no scales were applied to determine its severity. Despite these limitations, we believe that the results contribute to the knowledge of IgE sensitization in CRS and allows us to identify new questions that help direct future research.

In conclusion, sIgE plays an important role in the pathogenesis of CRS. The evaluation of atopy is a useful biomarker to identify endophenotypes and potentially for the establishment of specific allergen therapies.

## Abbreviations

ASA, Acetyl-salicylic acid; CRS, Chronic rhinosinusitis; NCT, Nasal challenge test; NERD, Non-steroidal anti-inflammatory (NSAID) drug-exacerbated respiratory disease; NSAID, Non-steroidal anti-inflammatory; sIgE, Specific IgE; SsIgE, Serum specific IgE; NsIgE, Nasa specific IgE

## Funding source

The study was supported by the Group of Clinical and Experimental Allergy, Hospital “Alma Mater de Antioquia” and the 10.13039/501100005278University of Antioquia (Medellín, Colombia).

## Data availability

The datasets generated during and/or analyzed during the current study are available from the corresponding author on reasonable request.

## Author contributions

All authors contributed to the study conception and design.

## Bioethical considerations

Permission was obtained from the technical and ethics committee of each participating center for the collection of information. The study protocol was approved by the institutional ethics committee (code IN48-2021 act #179, Hospital “Alma Mater de Antioquia”) and is in line with the Helsinki declaration. Each of the participants signed a informed consent.

## Authors’ consent for publication in WAO

The authors accept and confirm authorization to publish the article in question in the WAO Journal review.

## Declaration of competing interest

The authors declare no conflict of interest.
