# Supplementary material for: Role of specific immunoglobulin-E in chronic rhinosinusitis: Its clinical relevance according to nasal challenge test
Source: World Allergy Organ J. 2024 Oct 7;17(10):100953. doi: 10.1016/j.waojou.2024.100953 (PMC11491713; doi:10.1016/j.waojou.2024.100953)
Supplement: Multimedia component 1 [file mmc1.docx]

**eTable 1. General characteristics CRS group, rhinitis group and healthy group.**

| **Characteristics** | **Categories** | **Comparison groups** | | |  |
| --- | --- | --- | --- | --- | --- |
|  |  | **Global n= 174** | **Rhinitis group**  **n= 30** | **Healthy group**  **n= 30** | ***p*** |
| **Sex** | ***Female*** | 74 (42.5%) | 14 (46.6%) | 14 (46.6%) | 0.810 |
|  | ***Male*** | 100 (57.5%) | 16 (53.4%) | 16 (53.4%) |  |
| **Age group (years)** | | Me: 44 (RI: 13) | Me: 42 (RI: 16) | Me: 45 (RI: 15) | 0.790 |
| **Comorbidities** | ***Asthma*** | 63 (36.2%) | 6 (20%) | 0 | **<0.001** |
|  | ***AERD*** | 21 (12.1%) | 0 | 0 | **<0.001** |
|  | ***Polyps*** | 39 (22.4%) | 0 | 0 | **<0.001** |
|  | ***Anosmia*** | 18 (10.3%) | 0 | 0 | **<0.001** |
|  | ***Surgery for polyps*** | 17 (9.7%) | 0 | 0 | **<0.001** |
| **Symptoms** | ***SNOT22*** | Me: 24 (RI: 19) | No apply | No apply | No apply |
|  | ***TNSS*** | Me: 7 (RI: 4) | Me: 6 (RI: 4) | No apply | 0.790 |
| **Atopy (SPT)** | ***IgE monosensitization*** | 37 (21.2%) | 5 (16.6%) | 3 (10%) | **0.03** |
|  | ***IgE polysensitization*** | 55 (31.6%) | 25 (83.4%) | 3 (10%) |  |

**Table 1.** Sociodemographic characteristics of the patients. Atopy was defined as the presence of IgE in serum for at least one allergen. sIgE: specific IgE in serum. SnIgE: specific IgE in sinonasal mucus.

**eTable 2. Skin prick tests results**

| **Allergenic extract** | **Positive n (%)** |
| --- | --- |
| *Dermatophagoides pteronyssinus* | 92 (52.8%) |
| *Dermatophagoides farinae* | 89 (51.1%) |
| *Blomia tropicalis* | 25 (14.3%) |
| *Canis familiaris* | 22 (12.6%) |
| *Felix domesticus* | 16 (9.1%) |
| *Solenopsis invicta* | 7 (4%) |
| *Periplaneta americana* | 13 (7.4%) |
| *Blatella germanica* | 15 (8.6%) |
| *Culex pipenix* | 8 (4.5%) |
| *Alternaria alternata* | 6 (3.4%) |
| *Aspergillus fumigatus* | 12 (6.8%) |
| *Candida Albicans* | 9 (5.1%) |
| *Cladosporium herbarum* | 6 (3.4%) |
| *Grass group* | 14 (8%) |
| *Cereals group* | 6 (3.4%) |
| *Trees group* | 9 (5.1%) |
| *Flowers group* | 5 (2.8%) |
| *Cynodon dactylon* | 10 (5.7%) |
| *Poa pratense* | 6 (3.4%) |
| *Urtica dioca* | 2 (1.1%) |
| *Cupressus sempervirens* | 2 (1.1%) |
| *Pinus pinea* | 1(0.5%) |
| *Betula verrugosa* | 4 (2.2%) |
| *Zea mays* | 0 |
